# Morusin shows potent antitumor activity for melanoma through apoptosis induction and proliferation inhibition

**DOI:** 10.1186/s12885-023-11080-1

**Published:** 2023-06-29

**Authors:** Wei Liu, Yacong Ji, Feng Wang, Chongyang Li, Shaomin Shi, Ruochen Liu, Qian Li, Leiyang Guo, Yaling Liu, Hongjuan Cui

**Affiliations:** 1 Department of Dermatology, The Third Hospital of Hebei Medical University, Zi qiang Road 139, 050000 Shijiazhuang, China; 2 State Key Laboratory of Silkworm Genome Biology, Southwest University, No. 2 Tiansheng Road, Beibei District, 400715 Chongqing, P.R. China; 3 Cancer Centre, Reproductive Medicine Centre, Medical Research Institute, Southwest University, Chongqing, China; 4 Department of Pathology, School of Basic Medical Sciences, Fudan University, Shanghai, China; 5 The Ninth People’s Hospital of Chongqing, Affiliated Hospital of Southwest University, Chongqing, China

**Keywords:** Morusin, Melanoma, p53, Proliferation, Apoptosis, Metastasis

## Abstract

**Background:**

The discovery of new anti-melanoma drugs with low side effect is urgently required in the clinic. Recent studies showed that morusin, a flavonoid compound isolated from the root bark of Morus Alba, has the potential to treat multiple types of cancers, including breast cancer, gastric cancer, and prostate cancer. However, the anti-cancer effect of morusin on melanoma cells has not been investigated.

**Methods:**

We analyzed the effects of morusin on the proliferation, cell cycle, apoptosis, cell migration and invasion ability of melanoma cells A375 and MV3, and further explored the effects of morusin on tumor formation of melanoma cell. Finally, the effects of morusin on the proliferation, cycle, apoptosis, migration and invasion of A375 cells after knockdown of p53 were detected.

**Results:**

Morusin effectively inhibits the proliferation of melanoma cells and induces cell cycle arrest in the G2/M phase. Consistently, CyclinB1 and CDK1 that involved in the G2/M phase transition were down-regulated upon morusin treatment, which may be caused by the up-regulation of p53 and p21. In addition, morusin induces cell apoptosis and inhibits migration of melanoma cells, which correlated with the changes in the expression of the associated molecules including PARP, Caspase3, E-Cadherin and Vimentin. Moreover, morusin inhibits tumor growth in vivo with little side effect on the tumor-burden mice. Finally, p53 knockdown partially reversed morusin-mediated cell proliferation inhibition, cell cycle arrest, apoptosis, and metastasis.

**Conclusion:**

Collectively, our study expanded the spectrum of the anti-cancer activity of morusin and guaranteed the clinical use of the drug for melanoma treatment.

**Supplementary Information:**

The online version contains supplementary material available at 10.1186/s12885-023-11080-1.

## Background

In the past few decades, the incidence of melanoma has been increasing [1]. Melanoma is a skin cancer with a high mortality rate. Although the incidence of melanoma only accounts for about 3% of all malignant tumors, due to its rapid progression, strong lymphatic and distant metastasis ability [2–4] and poor prognosis, its mortality accounts for 90% of the mortality of skin tumors [5], and the survival time of patients with systemic metastasis is less than 1 year [4]. Current treatments include surgical excision, drug treatment, chemotherapy, targeted therapy and immunotherapy [6]. However, most of the anti-melanoma drugs commonly used in clinical practice have serious adverse reactions. Therefore, the development of new anti-melanoma drugs is an urgent problem at the present stage.

In recent years, it has been discovered that natural compounds, such as flavonoids, polyphenols, and alkaloids [7], have good killing effects on tumor cells, while little effect on normal cells and few adverse reactions [6]. Luteolin (3,4,5,7-tetrahydroxy flavone) is a flavonoid found in different plants, such as vegetables and fruits [8]. Studies have found that it has anti-inflammatory, anti-allergic, anti-anxiety, neuroprotective and anti-cancer effects [8–10]. Luteolin inhibits the proliferation [11] and induces the apoptosis of A375, human melanoma cells, by reducing the expressions of MMP-2 and MMP-9 through the PI3K/AKT pathway [12]. In addition, luteolin significantly inhibits the migration, invasion, adhesion and microtubule formation potential of highly metastatic A375 and B-16-F10 melanoma cells with no significant cytotoxicity [13]. Resveratrol, a polyphenol component, is found in grapes and some other plants. It has anti-cancer properties [14] and can significantly inhibit the proliferation of melanoma cells [15, 16], induce apoptosis by up-regulating p53 in a concentration-dependent manner [16], inhibit cell migration [17] and murine melanoma tumor growth [18]. Berberine is an isoquinoline alkaloid present in several plants, and it has anti-cancer activity and less toxic to normal cells [19]. Berberine can induce cell morphological changes, inhibit migration and invasion of A375 cells [20]. Morusin is one of the isoprene flavonoid derivatives from the root bark of Morus (Fig. S1A). Recent studies have reported that morusin exhibits anti-tumor biological activity in many human cancers. In human gastric cancer, morusin inhibits cell proliferation and tumor growth by down-regulating c-Myc [21]. Morusin induces autophagy by activation of AMPK and inhibition of mTOR activity in HeLa cells [22], and induces apoptosis in human colorectal cancer cells through NF-κB pathway [23]. Morusin could inhibit renal carcinoma cell growth and migration, induce cell apoptosis through the mitogen-activated protein kinase (MAPK) signaling pathway [24]. In human non-small cell lung cancer and human hepatocellular carcinoma, morusin can induce apoptosis through EGFR/STAT3 or IL-6/STAT3 signaling pathway to play an anti-cancer effect [25, 26].

As we all know, p53 is a transcription factor that regulates many cell pathways, such as cell cycle, DNA damage, apoptosis, autophagy and cell metabolism. Loss of p53 expression can down-regulate BubR1, damage part of the function of the spindle checkpoint, and lead to the occurrence and development of colorectal cancer [27]. In addition, p53 not only plays an important role in cancer, but also has a regulatory function in lung fibrosis and adipogenesis [28, 29]. Due to its more functions, p53 is considered an important target for the development of new anti-cancer treatment strategies.

Our study is the first to demonstrate the anti-tumor effect of morusin in melanoma, which inhibits cell proliferation, induces cell cycle arrest, promotes apoptosis, and inhibits cell migration and invasion by increasing the expression of p53. These results illustrate the anti-melanoma effect of morusin and the underlying mechanism, suggesting potential clinical usage of the drug.

## Methods

### Cell culture

The human melanoma cell lines (A375 and MV3), PIG1 melanocytes, and the human embryonic renal cell line 293FT were purchased in American Type Culture Collection (ATCC, Manassas, VA, USA). HaCaT keratinocytes were purchased from Cell Line Service GmbH. These cells were stored in our laboratory. A375, PIG1, and HaCaT cells were cultured in Dulbecco’s modified Eagle’s medium (DMEM, Gibco, New York, NY, USA). Roswell Park Memorial Institute-1640 (RPMI-1640, Gibco) was used for maintaining MV3 cells. The 293FT cells were cultured as described previously [30]. They were supplemented with 10% fetal bovine serum (FBS, Gibco), 1% penicillin and streptomycin (P/S, Invitrogen, Califonia, CA, USA). All cells were cultured at 37℃ in a humidified incubator with 5% CO_2_.

### Morusin treatment

Morusin was purchased form Must Bio-Technology Co., Ltd. (Cat. No.:62596–29-6; Chengdu, China) and then dissolved in DMSO as 100 mM stock solution. The human melanoma cell lines, A375 and MV3 were treated with morusin in different concentrations (2, 5 and 10 μΜ were used in A375 cells, and 5, 10 and 15 μΜ were used in MV3 cells). In our experiments, DMSO was used as control group. Maintained for different times (0, 12, 24 and 36 h), A375 cells were treated with 5 μΜ morusin, and MV3 cells were treated with 10 μΜ morusin. Each experiment was repeated three times.

### Transfection and infection

The plasmid shp53(1#, 3#) and shGFP were purchased from Sigma-Aldrich as described previously [30]. According to manufacturer’s instructions, first, liposomes and packaging plasmids (PLP1, PLP2, VSVG, target plasmids: shGFP, shp53 1#, shp53 3#) were transfected into 293FT cells. After 8 h, the old medium was removed, and fresh 293FT medium was added. After 48 h, the viral supernatant was collected and used to infect the melanoma cell line A375. Then the positive cells were screened by puromycin.

### Cell viability assays

The IC50 of melanoma cells A375 and MV3, as well as PIG1 and HaCaT cells, was determined by MTT assay. By using the hemocytometer, the number of cells in each well of 96-well cell culture plate was 1000. After incubating with different doses of morusin (0.001、0.01、0.1、1、10、20、40、60、80 and 160 μΜ) or DMSO for 24 h, 20 μL of MTT (5 mg/mL, Sigma Aldrich, USA) staining solution was added into each well, which was put back to 37℃ and cultured in 5% CO_2_ incubator. After 1 h, the old medium was removed. And 200 μL of DMSO was added into each well to determine the absorbance at 490 nm by the Thermo Science Microplate Reader. We analyzed the data through GraphPad Prism 8.3.0. Each experiment was repeated three times.

### BrdU staining

The number of cells per well was 4 × 10^4^ in the 24-well plate, and the cells were cultured in 500 μL medium (DMEM for A375 and RPMI-1640 for MV3) for 12 h in the cell culture chamber. Then morusin (5 μΜ of morusin was added to A375, 10 μΜ of morusin was added to MV3) was added to medium, and DMSO was added to the control group for 24 h. The detailed steps of the following experimental methods were described in the previous article [31]. Finally, photos were taken under the microscope and at least 10 randomly selected regions were analyzed for BrdU positive cells.

### Flow cytometry analysis

After A375 cells were treated with 2 or 5 μΜ morusin, MV3 cells were treated with 5 or 10 μΜ morusin (equal volume of DMSO was used to control) for 24 h, the cells were washed 3 times with pre-cooling PBS. For the cell cycle assay, the cells were suspended in 75% ethanol at 4℃ for 24 h. After that, cells were washed with PBS buffer, then incubated with propidium iodide (PI) containing with RNase A (Sigma Aldrich, USA) at 37℃ for 1 h in dark. Cells were collected by the FACS C6 (BD, USA) and the data was analyzed with Cell Quest software. For the cell apoptosis assay, the cells were resuspended in 100 μL binding buffer with 5 μL Annexin V and 5 μL propidium iodide (PI), culturing for 30 min [32]. Then the cells were collected by the FACS C6 and the data was analyzed by using the Cell Quest software. Each experiment was repeated three times.

### Western blot analysis

A375 and MV3 cells were collected and then lysed with RIPA lysis buffer, which contained phenyl methane sulfonyl fluoride (PMSF). Then BCA protein assay kit (Beyotime Biotech, China) was used to measure protein concentration. The proteins were separated by 10% or 12% SDS-PAGE, and then transferred to a polyvinylidene difluoride membrane. After blocking with 5% defatted milk for 2 h, the membrane was cut according to the size of marker. The membrane was incubated with primary antibody against p53 (1:1000, Proteintech, China, 10,442–1-AP), p21 (1:1000, Proteintech, China, 10,355–1-AP), CyclinB1 (1:1000, Cell Signaling Technology, CST, USA, #12,231), CDK1 (1:2000, Abcam, USA, ab245318), PARP (1:1000, Cell Signaling Technology, CST, USA, #9542), Caspase3 (1:1000, Cell Signaling Technology, CST, USA, #9662), MMP2 (1:1000, Proteintech, China, 10,373–2-AP), E-Cadherin (1:1000, Proteintech, USA, 20,874–1-AP), Vimentin (1:1000, Proteintech, USA, Catalog number: 10366–1-AP), Tubulin (1:1000, Cell Signaling Technology, CST, USA, #2144) at 4℃ overnight. After washing with TBST buffer for 3 times, the membrane was incubated with HRP-conjugated secondary antibody (goat anti-rabbit IgG and goat anti-mouse IgG, 1:10,000, Life Technology, USA) for 2 h at room temperature. Then the proteins bands were finally visualized and captured by the detection analysis system (Clinx Science, China). Unfortunately, we lack images of sufficient length.

### Wound-healing assays

The detailed experimental method has been reported in the previous literature [33–35]. In brief, 1 × 10^6^ cells were planted in each well of the 6-well plate. When the cells were overgrown, we scratched them by the tip of a white spear and washed them with PBS. Finally, serum-free medium with morusin (A375 cells were used 2 and 5 μΜ, MV3 cells were used 5 and 10 μΜ) or DMSO was added to cells. Observed the migration of cells in the exposed area and took pictures at indicated time.

### Cell migration and invasion

Briefly, a total of 5 × 10^5^ cells were added into multiporouspolycarbonate membrane insert (8-μm pore size) (Corning), which were fitted into a 24-well plate for cell migration assays. Added 200 μL 1% serum medium to the inside and 500 μL 10% FBS medium to the outside as the infiltrating agent. DMEM medium contained 5 μΜ morusin or DMSO. 1640 medium contained 10 μΜ morusin or DMSO. After the 24-well plate was placed in an incubator at 37℃ (A375 cells were cultured for 12 h, MV3 cells were cultured for 24 h), the chamber was washed with PBS solution 3 times, and 4% paraformaldehyde was added to fix it for 20 min. Washed once with PBS solution and put it into 1 mL crystal violet staining solution for 20 min. After gently wiping the bottom with cotton, we observed and took pictures with the microscope. The transwell invasion assays were performed under the same conditions as the transwell migration assays, but the top chamber was added 50 μL matrigel (10 mg/mL, Corning), then incubated for 72 h. Each experiment was repeated three times.

### Soft agar colony formation assay

The effect of morusin on the colony formation ability of melanoma cells was determined by soft agar assay. In short, 2 × DMEM/RPMI 1640 medium (DMEM medium for A375 cells, and RPMI 1640 medium for MV3) was mixed with 1.2% low-melting-point agarose in a 1:1 ratio to make the base agar, and added 1 mL to a 6-well plate. 1000 of A375 or MV3 cells were mixed with 1.5 mL DMEM/RPMI 1640 medium containing 0.3% agarose with morusin (5 μΜ, 10 μΜ or DMSO) and added to the top of base agar. Generally, after 3 weeks, the images were collected by white light microscope. Then 200 μL MTT staining solution was added into each well. Finally, imaging and counting were performed after the reaction at 37℃ for 30 min.

### Animal studies

Animal experiments were approved by the animal ethics committee of Southwest University (Chongqing, China). Three-week-old female mice (NOD/SCID) were purchased and housed in SPF room for 1 week before injection. Then 1 × 10^6^ MV3 cells in 100 μL PBS were subcutaneously injected to flanks of mice. After one week, the mice were randomly divided into two groups. One group was intraperitoneally injected with morusin (25 mg/kg) and another group was injected with DMSO as control every 2 days for 20 days. In the meantime, the mice weight and tumor volume (tumor volume = length × width^2^ × (π/6)) were measured every 2 days. After three weeks, the tumors were collected and fixed with paraformaldehyde. Then hematoxylin–eosin (H&E) and immunohistochemistry (IHC) experiments were performed. Finally, we injected Avertin and euthanized the mice by cervical dislocation.

### Vector construction and transfection

pLKO.1 expressing p53 short hairpin RNA (shp53) were constructed by using the double strand of shRNAs below. shp53 1#: F: CAGCACATGACGGAGGTTGT; R: TCATCCAAATACTCCACACGC.shp53 3#: F: ACAGCTTTGAGGTGCGTGTTT; R: CCCTTTCTTGCGGAGATTCTCT.

### Quantitative real-time PCR (qRT-PCR)

Cells were collected after treatment with DMSO or morusin at 37 °C for 24 h. Total RNA was isolated from the cells using TRIzol reagent (Invitrogen, Carlsbad, CA, USA) according to the manufacturer’s instructions. Reverse transcription was performed using M-MLV reverse transcriptase (Promega, Madison, WI, USA). Quantitative real-time fluorescent quantitative PCR (qRT-PCR) was used to detect the relative expression of the p53 gene.

### Statistics analysis

All experiments were confirmed by three independent experiments. All data were analysed by GraphPad Prism 8.3.0. In this study, quantitative data were expressed as the mean ± standard deviation. One-way ANOVA was used to assess the mean difference among three or more groups, further multiple comparisons were done with Tukey’s test, and two-tailed Student’s t-test was performed for paired samples, *P* < 0.05 was considered as statistically significant (*P < 0.05, **P < 0.01, ***P < 0.001).

## Results

### Morusin inhibits cell proliferation in melanoma cells

**Fig. 1:**
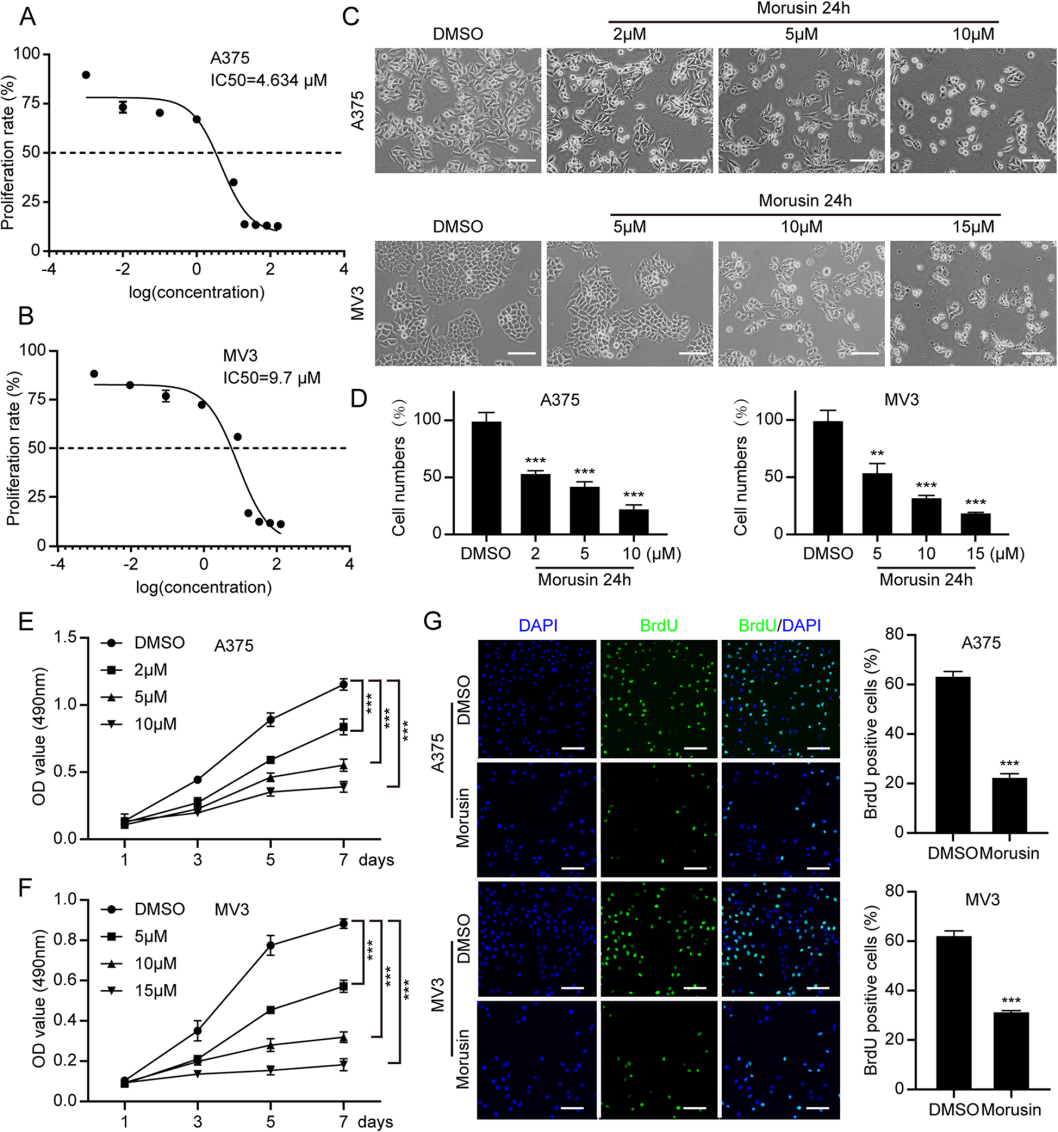
Morusin inhibits cell growth and proliferation in melanoma cells. The IC50 of **A** A375 and **B** MV3 cells. **C** The cell morphology of A375 and MV3 cells after treating with indicated concentration of morusin for 24 h. DMSO was used as control. Scale bar was 100 μm. **D** The cell numbers of A375 and MV3 were counted and displayed. The DMSO treatment group was considered to be 100%. The viability of **E** A375 and **F** MV3 after treating with DMSO or morusin. **G** The image and quantification of BrdU staining cells of A375 and MV3 after treating with DMSO or morusin (5 μΜ for A375 and 10 μΜ for MV3) for 24 h. Scale bar was 100 μm. Each experiment was repeated three times. All data were shown as the mean ± SD and analyzed by two-tailed Student’s t-test. *P < 0.05, **P < 0.01, ***P < 0.001

In order to explore the viability effect of morusin in melanoma cells, we performed IC50 assay by 10 different doses of morusin treatment for 24 h in A375 and MV3 cells, and the results showed that the IC50 of morusin in A375 is 4.634 μΜ and MV3 is 9.7 μΜ (Fig. 1A and B). Meanwhile, the IC50 of morusin for PIG1 and HaCaT cells were 21.04 and 18.01 μM, which were higher than that of melanoma cells (Fig. S1C and D). Next, the human melanoma A375 and MV3 cells were treated with different concentrations of morusin for 24 h. By the microscope, we found that the obvious changes in the morphology of the cells after morusin treatment, and the number of cells decreased in a dose-dependent manner as well (Fig. 1C and D). MTT assay also showed that comparing with DMSO group, A375 cells treated with 5 and 10 μΜ, MV3 cells treated with 10 and 15 μΜ morusin had sharp decline in cell growth curve (Fig. 1E and F). Besides, 10 μM morusin has no significant effect on cell viability of PIG1 and HaCaT cells (Fig. S1E and F). Dacarbazine (DTIC) is the first generation of drug approved by the US FDA for the treatment of melanoma, and its therapeutic effect is obvious. In this experiment, we examined the change in cell viability after 24 h treatment of A375 and MV3 cells with different concentrations of DTIC (CAS: 4342–03-4; Shanghai, China), and the results showed that the IC50 of A375 cells was 81.9 μM (Fig. S1J) and the IC50 of MV3 cells was 84.7 μM (Fig. S1K). In the MTT experiment, we can see that the growth curve of A375 cells decrease more slowly after 5 μΜ morusin treatment than after 80 μΜ DTIC treatment (Fig. S1L). Similarly, the same results can be obtained after 10 μΜ morusin treatment and 85 μΜ DTIC treatment of MV3 cells (Fig. S1M). BrdU staining assay showed that morusin remarkably decreased the percentage of BrdU-positive cells, compared with DMSO-treated cells in A375 and MV3 (Fig. 1G). These results suggested that morusin had a significant inhibitory effect on the cell proliferation in melanoma cells.

### Morusin induces cell cycle arrest at G2/M phase in melanoma cells

**Fig. 2:**
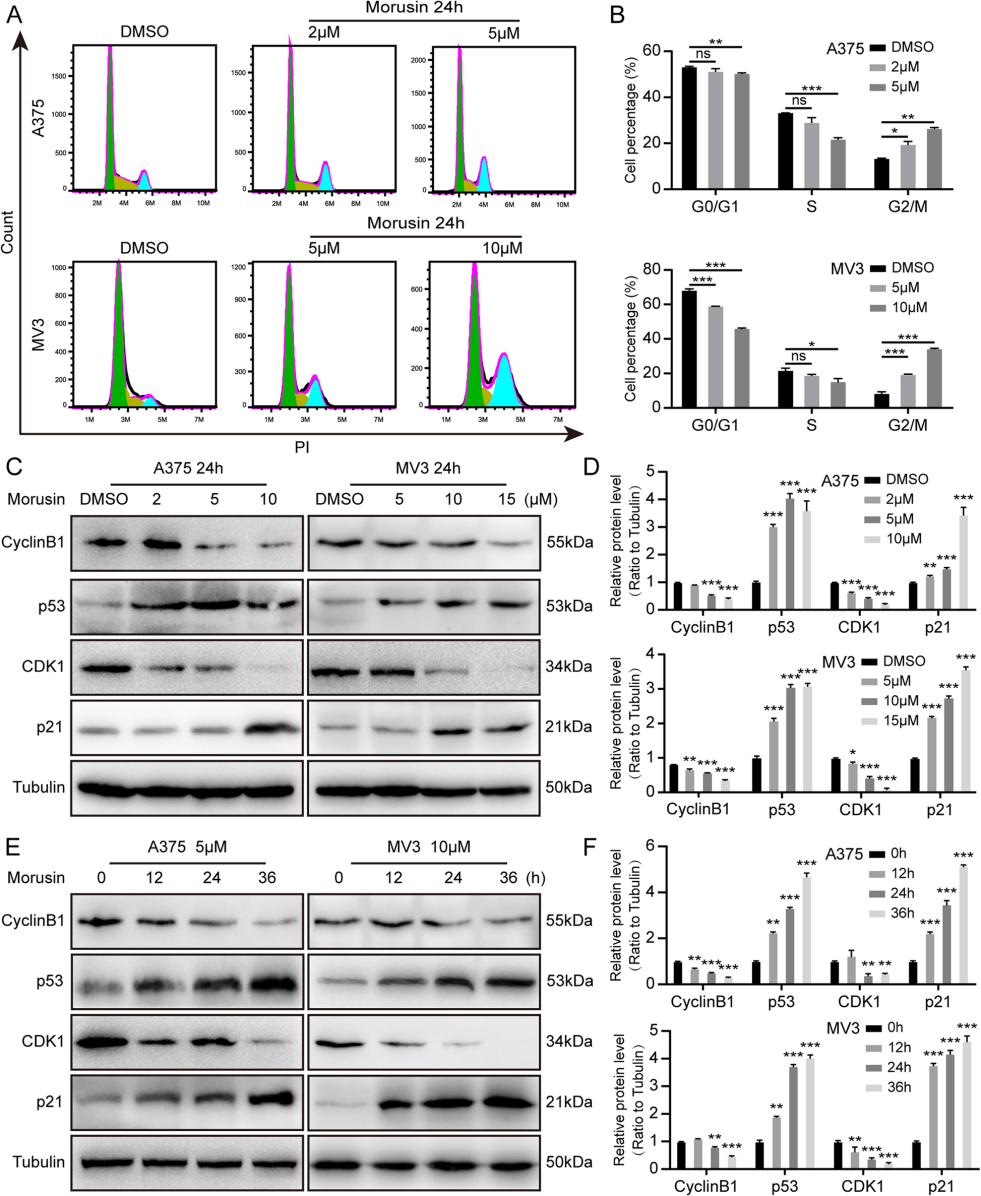
Morusin induces cell cycle arrest at G2/M phase in melanoma cells. **A** The cell cycle of A375 and MV3 cells treated with DMSO or morusin (5 μΜ for A375 and 10 μΜ for MV3) for 24 h was analyzed by flow cytometry. **B** Percentage of indicated A375 and MV3 cells in different periods. **C** The expression levels of CyclinB1, p53, p21 and CDK1 in A375 and MV3 cells treated with morusin in different concentrations (2, 5, 10 μΜ for A375 and 5, 10, 15 μΜ for MV3) or DMSO for 24 h were determined by Western blot analysis. Tubulin was used as a control. **D** Densitometry of western blot in panel C. **E** The expression levels of CyclinB1, p53, p21 and CDK1 in A375 and MV3 cells treated with morusin (5 μΜ for A375 and 10 μΜ for MV3) in different times for 0, 12, 24, 36 h. Tubulin was used as a control. **F** Densitometry of western blot in panel E. Each experiment was repeated three times. All data were shown as the mean ± SD and analyzed by two-tailed Student’s t-test. *P < 0.05, **P < 0.01, ***P < 0.001

Cell proliferation is closely related to the cell cycle. Next, we further explored whether morusin has effect on the cell cycle. The cell cycle was detected by PI single-staining flow cytometry. The results showed that comparing with the control group, the percentage of G2/M phase cells was significantly increased after treating with 2 and 5 μM morusin on A375 cells, 5 and 10 μM morusin on MV3 cells for 24 h. The difference was statistically significant (*P* < 0.05) (Fig. 2A and B). Western blot experiment further verified the expression levels of related cyclins. The results showed that, after treatment with morusin, the protein expression levels of p53 and p21 were increased in a concentration-dependent manner (A375 cells were treated with DMSO, 2 μM, 5 μM, 10 μM, and the doses treated on MV3 cells were DMSO, 5 μM, 10 μM, 15 μM) and time-dependent manner (0 h, 12 h, 24 h, 36 h). In the meantime, CyclinB1 and CDK1 was down-regulated (Fig. 2C-F). Above results indicated that morusin induces cell cycle arrest at G2/M phase, thereby inhibiting the proliferation of melanoma cells.

### Morusin induces apoptosis in melanoma cells

**Fig. 3:**
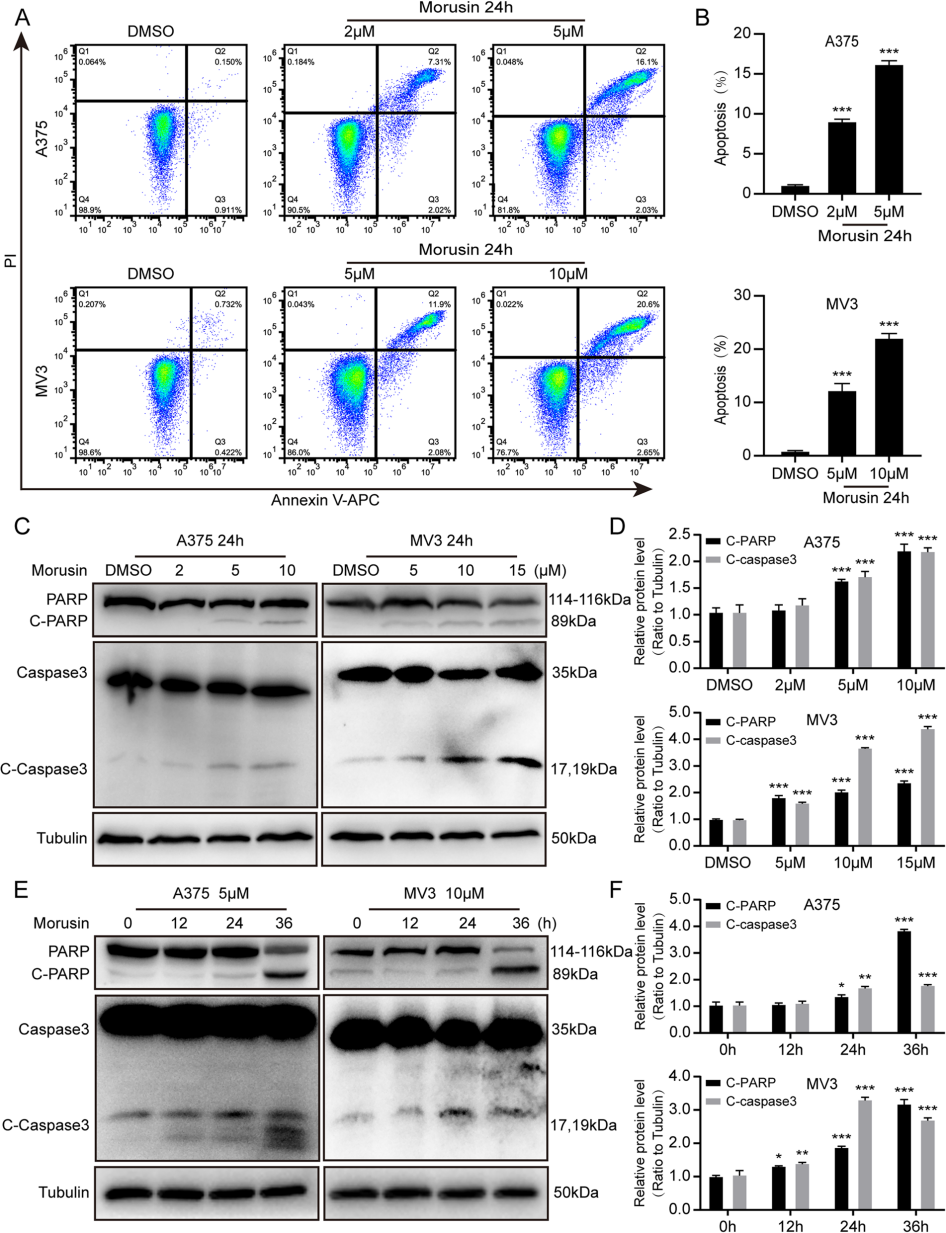
Morusin induces apoptosis in melanoma cells. **A** Apoptosis of A375 and MV3 cells treated with DMSO or morusin (2, 5 μΜ for A375 and 5, 10 μΜ for MV3) for 24 h was analyzed by flow cytometry. **B** Apoptosis rate of melanoma cells in Panel A. **C** The expression levels of PARP and Caspase3 in A375 and MV3 cells treated with morusin in different concentrations (2, 5, 10 μΜ for A375 and 5, 10, 15 μΜ for MV3) or DMSO for 24 h were determined by Western blot analysis. Tubulin was used as a control. **D** Densitometry of western blot in panel C. **E** The expression levels of PARP and Caspase3 in A375 and MV3 cells treated with morusin (5 μΜ for A375 and 10 μΜ for MV3) in different times for 0, 12, 24, 36 h. Tubulin was used as a control. **F** Densitometry of western blot in panel E. Each experiment was repeated three times. All data were shown as the mean ± SD and analyzed by two-tailed Student’s t-test. *P < 0.05, **P < 0.01, ***P < 0.001

In order to explore whether morusin can also induce apoptosis, we used AnnexinV-APC and PI for flow cytometry assay. As shown in Fig. 3A and B, there was obvious apoptosis after morusin treatment. To further certify this result, we treated A375 cells with 0, 2, 5 and 10 μΜ, MV3 cells with 0, 5, 10 and 15 μΜ morusin for 24 h. Western blot assay results showed that Cleaved-PARP (C-PARP) and Cleaved-Caspase3 (C-Caspase3) increased in a dose-dependent manner (Fig. 3C and D). Besides, we also found that the levels of C-PARP and C-Caspase3 were increased in a time-dependent manner (Fig. 3E and F). All the results suggested that morusin induces apoptosis in melanoma cells.

### Morusin inhibits cell migration and invasion in melanoma cells

**Fig. 4:**
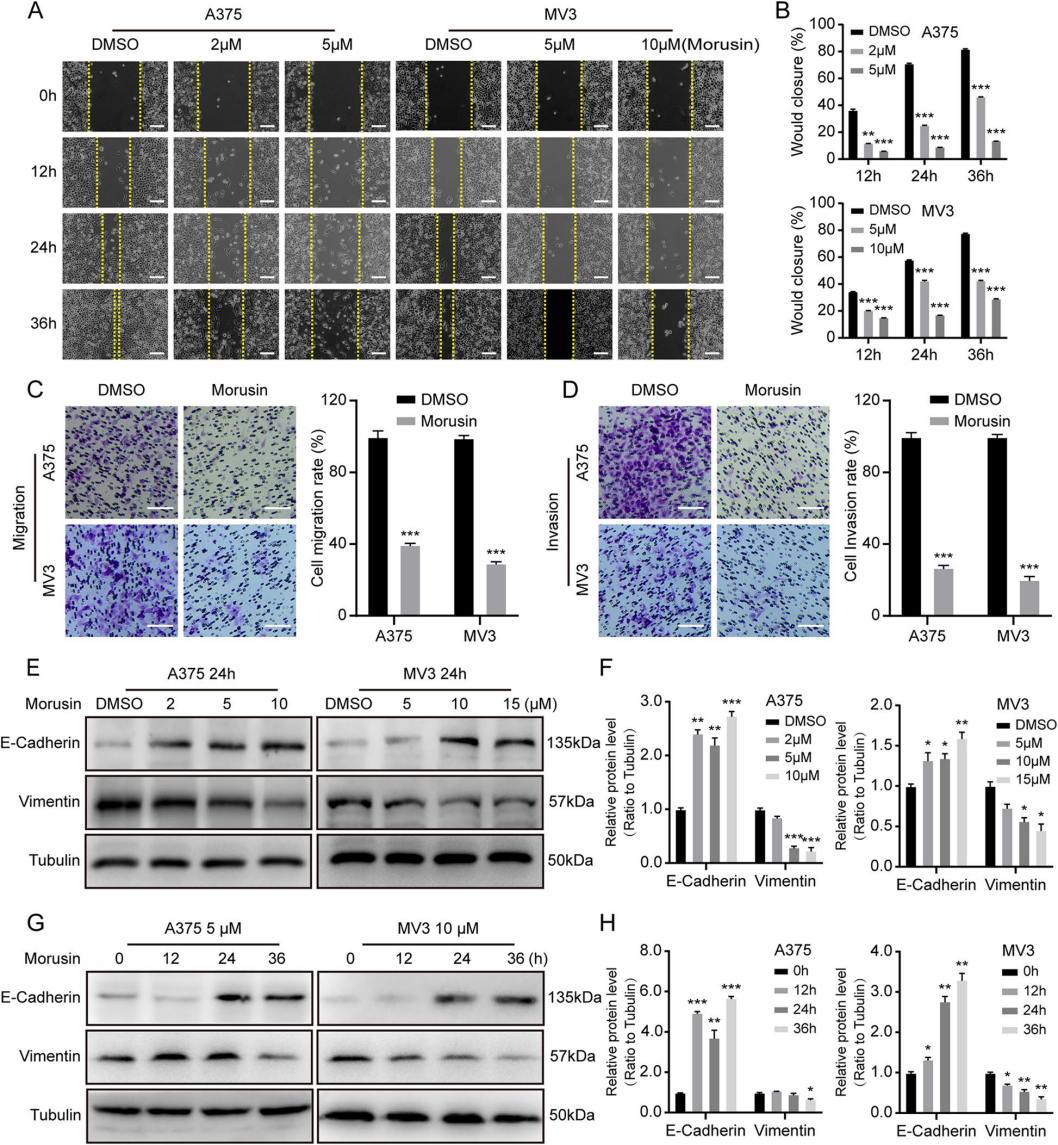
Morusin inhibits cell migration and invasion in melanoma cells. **A** The migration of A375 and MV3 cells treated with morusin for the corresponding time was measured by wound-healing assay. Scale bar was 100 μm. **B** The effect of morusin on the healing of melanoma cells. The healing rate of 0 h was considered to be 100%. **C** Transwell migration assays of A375 and MV3 cells treated with DMSO or morusin (5 μΜ for A375 and 10 μΜ for MV3) for 24 h. Scale bar was 100 μm. The statistical analysis was presented in histograms, and cell migration rates were normalized by proliferation. **D** Transwell invasion assays of A375 and MV3 cells treated with DMSO or morusin (5 μΜ for A375 and 10 μΜ for MV3) for 72 h. Scale bar was 100 μm. Cell invasion rates were normalized by proliferation. **E** The expression levels of E-Cadherin and Vimentin in A375 and MV3 cells treated with morusin in different concentrations (2, 5,10 μΜ for A375 and 5, 10, 15 μΜ for MV3) or DMSO for 24 h. **F** Densitometry of western blot in panel E. **G** The expression levels of E-Cadherin and Vimentin in A375 and MV3 cells treated with morusin (5 μΜ for A375 and 10 μΜ for MV3) in different times for 0, 12, 24, 36 h. Tubulin was used as the control. **H** Densitometry of Western blot in panel G. Each experiment was repeated three times. All data were shown as the mean ± SD and analyzed by two-tailed Student’s t-test. *P < 0.05, **P < 0.01, ***P < 0.001

Melanoma cells have the superior ability of migration and invasion. In order to explore the effect of morusin on the migration and invasion ability of melanoma cells, we conducted wound healing assay, transwell migration and invasion assays and Western blot experiment. Wound healing assay showed that melanoma cells treated with morusin had a significantly weaker ability to heal scratches than the control group (Fig. 4A and B). Transwell migration assay also revealed that the migration ability of the melanoma cells treated with morusin was significantly reduced comparing with the control group (Fig. 4C). Similarly, the result of invasion assay showed that the number of cells crossed the matrigel was significantly reduced after morusin-treated comparing with control group (Fig. 4D). Western blot assay indicated that morusin up-regulated E-Cadherin and down-regulated Vimentin expression in a dose-dependent and time-dependent manner (Fig. 4E-H). And we found that the expression of MMP2 was also down-regulated with the concentration and time of morusin (Fig. S1G and H). To sum up, these findings demonstrated that morusin inhibits cell migration and invasion in melanoma cells.

### *Morusin inhibits tumor growth both *in vitro* and *in vivo

**Fig. 5:**
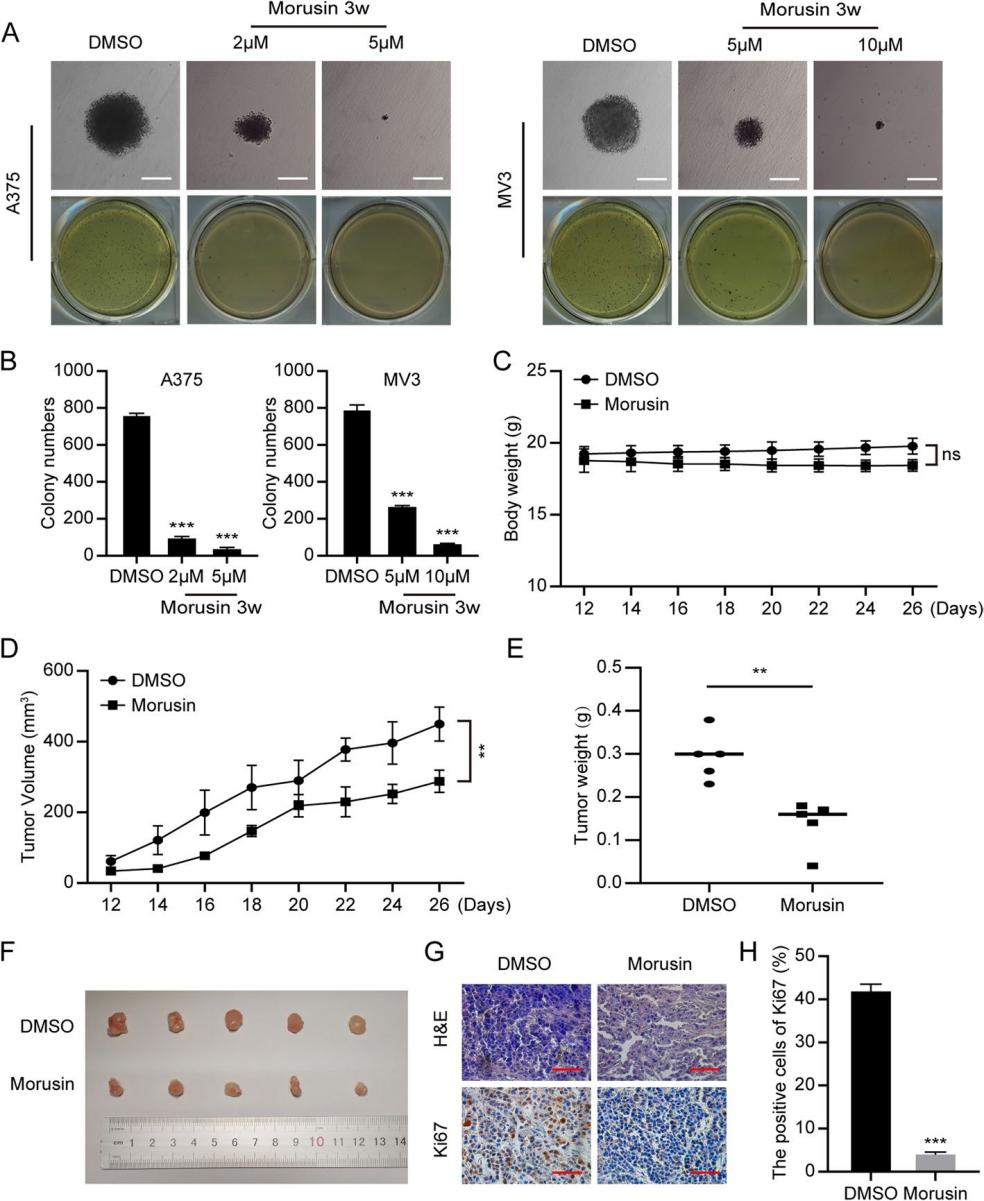
Morusin inhibits tumor growth in vitro and in vivo*.* **A** The clone formation ability of A375 and MV3 after treating with 5 and 10 μΜ morusin was detected by soft agar assay. Scale bar was 100 μm. **B** Colony numbers in panel A were quantified. **C** Body weight of mice treated with morusin or DMSO. **D** Tumor volume of mice treated with morusin (25 mg/kg) or DMSO. **E** Tumor weight of mice treated with morusin (25 mg/kg) or DMSO. **F** Photograph of tumors from indicated mice. **G** H&E and IHC staining of Ki67 in indicated tumors. Scale bar was 100 μm. **H** The positive cells of Ki67 in panel G. All data were shown as the mean ± SD and analyzed by two-tailed Student’s t-test. *P < 0.05, **P < 0.01, ***P < 0.001

To further assess the effects of morusin in colony formation, we used the melanoma A375 and MV3 cells as the research objects to conduct soft agar clone formation experiment. The inverted microscope showed that comparing with the control group, the size of single clone in the morusin treatment groups was significantly smaller, and the number of clones formed was significantly reduced (Fig. 5A and B). Next, MV3 cells were injected subcutaneously into four-week-old mice, and the growth status of the mice were observed every two days. It was observed that there was no significant difference between the body weights of the experimental group and the control group (Fig. 5C). The tumor volume of the morusin treatment group was lower than the control group (Fig. 5D). And the tumor weight and size of the morusin treatment group were obviously smaller than the control group (Fig. 5E and F). At the same time, the hematoxylin–eosin (H&E) results showed that comparing with the control group, the number of cells in the experimental group was reduced and the nucleus became smaller, and the immunohistochemistry (IHC) staining results suggested that the expressions of Ki67, CDK1 and CyclinB1 were significantly reduced, while the expressions of p53 and p21 were increased in the morusin-treated group (Fig. 5G and H) (Fig. S1N). The data indicated morusin could inhibit the colonization of melanoma cells and inhibit tumor formation in mice.

### Morusin-inducing cell proliferation inhibition, cell cycle arrest, apoptosis, and metastasis can be partially recovered by knocking down the p53

**Fig. 6:**
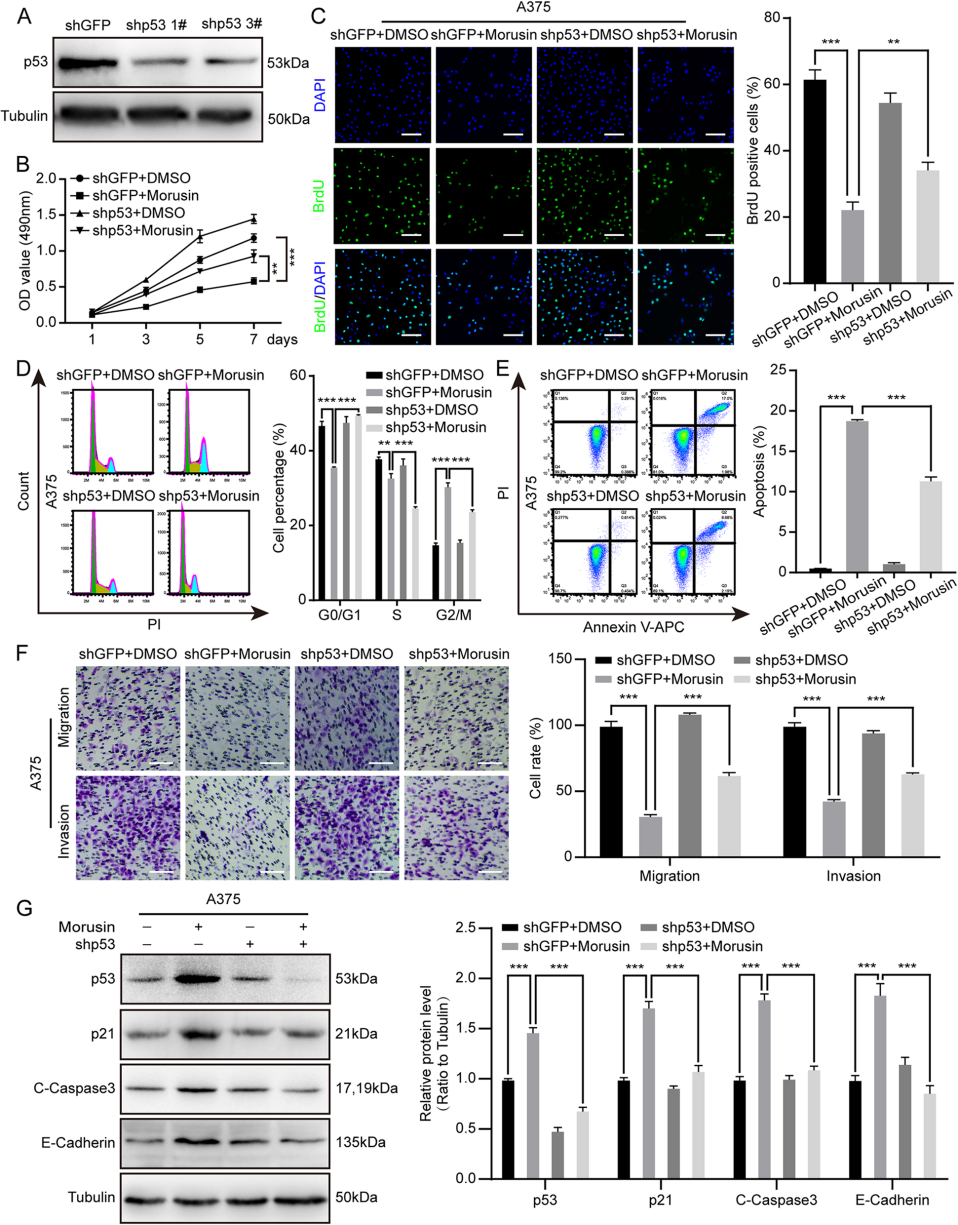
Morusin-inducing cell proliferation inhibition, cell cycle arrest, apoptosis, and metastasis can be recovered by knocking down p53. **A** The expression level of p53 was detected by Western blot in A375 cells. **B** Growth curve of knock down p53 after treating with 5 μΜ morusin in A375 cells. DMSO and shGFP were used as control. **C** The image and quantification of BrdU positive cells in knockdown p53 A375 cells treatment with 5 μΜ morusin for 24 h. Scale bar was 100 μm. **D** Cell cycle was detected in knockdown p53 A375 cells after treating with 5 μΜ morusin for 24 h, and percentage of A375 cells in different phase. DMSO and shGFP were used as control. **E** Apoptosis of knockdown p53 A375 cells treated with 5 μΜ morusin for 24 h was analyzed by flow cytometry, and apoptosis rate of melanoma cells was quantified. DMSO and shGFP were used as control. **F** Transwell migration and invasion assays of knockdown p53 A375 cells treated with 5 μΜ morusin for 24 h and 72 h were analysed. Scale bar was 100 μm. DMSO and shGFP were used as control. Cell migration and invasion rates were normalized. **G** The expression of p53, p21, C-Caspase3 and E-Cadherin were checked in knockdown p53 A375 cells treated with 5 μΜ morusin for 24 h. DMSO and shGFP were used as control. Tubulin was used as the control. All data were shown as the mean ± SD and analyzed by two-tailed Student’s t-test. *P < 0.05, **P < 0.01, ***P < 0.001

As is known to all, p53 as a tumor suppressor can maintain normal development and tissue homeostasis [36]. Meanwhile, we found that the expression of p53 was remarkably increased in A375 and MV3 after treatment with morusin. Therefore, the expression of p53 in A375 and MV3 cells was detected by qRT-PCR. The results showed that morusin increased the mRNA expression of p53(Fig. S1B). In order to explore the role of p53 in our experiment, we successfully knocked down p53 in A375 cells (Fig. 6A). MTT assay showed that after treating with 5 μΜ morusin in A375 cells, the cell growth was partially recovered by knocking down p53 (Fig. 6B). At the same time, BrdU staining results indicated that knock down p53 could rescue the DNA synthesis reduced by morusin (Fig. 6C). To investigate whether knock down of p53 effect the cell cycle, we treated the shGFP and shp53 group with 5 μΜ morusin and then performed with flow cytometry analysis. The results showed that the percentage of G2/M phase in the shp53 group showed a slight decrease after treating with morusin for 24 h in A375 cells (Fig. 6D), which means knocking down p53 could rescue cell cycle arrest induced by morusin. Subsequently, we examined the effect on apoptosis. A375 cells were treated with 5 μΜ morusin for shp53 group and shGFP group. After 24 h, flow cytometry showed that comparing with the shGFP group, the shp53 group showed a decline in cells apoptosis (Fig. 6E), which indicated that knocking down p53 could alleviate apoptosis induced by morusin. In addition, the migration and invasion assay found that the number of cell migration and invasion increased in the shp53 group comparing with the shGFP group (Fig. 6F), suggesting that knocking down p53 could rescue the reduced ability of cell migration and invasion induced by morusin. Finally, Western blot results also showed that comparing with the shGFP group, the expression levels of p53, p21, C-Caspase3 and E-Cadherin were decreased (Fig. 6G), and the expression levels of CDK1 and CyclinB1 were increased (Fig. S1I) in shp53 group following morusin treatment. The above results indicated that morusin-inducing cell proliferation inhibition, cell cycle arrest, apoptosis, and metastasis could be partially recovered by knocking down p53.

## Discussion

Melanoma, a malignant tumor of the skin, is caused by the excessive proliferation of melanocytes, and its incidence is increasing [37]. Surgery combined with chemotherapy is the main treatment, but due to the strong invasion and metastasis ability of melanoma cells, the current treatment cannot achieve satisfactory therapeutic effect [38, 39]. Therefore, it is particularly important to explore new and efficient treatment methods.

Flavonoids, a kind of natural products, are widely found in the plant kingdom. They not only have anti-inflammatory, antioxidant, antiviral and neuroprotective effects [40], but also have a killing effect on tumor cells. Morusin is one of the important flavonoids. Recent studies showed that morusin had anti-cancer activity in many human cancers, such as breast cancer [41], pancreatic cancer [42], lung cancer [43, 44] and other tumor types [21, 22, 45]. In addition, Morusin ameliorates IL-1β-Induced chondrocyte inflammation and osteoarthritis via NF-κB signal pathway [46]. Meanwhile, as an anticancer candidate, morusin exerts antitumor effects in hepatocellular cancers through AMPK-mediated G1 arrest and anti-glycolysis [47]. Still, the anti-tumor activity of morusin in human melanoma is unclear. In this study, we found, for the first time, that morusin has anti-melanoma effect. We used different concentrations of morusin to treat human melanoma cells A375 and MV3. The MTT assays indicated that morusin inhibits cell proliferation in a dose-dependent and time-dependent manner. Additionally, we found that the IC50 of morusin is significantly high in the normal PIG1 and HaCaT cells, then treating PIG1 and HaCaT with IC50 of morusin in MV3 (10 μM), which is the highest concentration used in cancer cells in this study, showed no obvious toxicity. Dacarbazine (DTIC) is the first generation of drugs approved by the US FDA for the treatment of melanoma, and its therapeutic effect is obvious, and many scholars have studied the efficacy of dacarbazine [48, 49]. In our experiments, we can see that 80 μM DTIC has a significant inhibitory effect on the growth of A375 cells. At the same time, 5 μM morusin has a better inhibitory effect on A375 cells. The same result was found in MV3 cells. Of course, this result needs to be verified in the clinic in the future. BrdU assays also showed a remarkable decrease in the percentage of BrdU-positive cells which were treated with morusin. The soft agar assays suggested that after treatment with morusin, the colonies were lesser and smaller than the DMSO group. In vivo, xenograft experiment showed that the tumor volume and weight were significant reduced. All evidence above indicated that morusin could inhibit the growth of melanoma cells in vitro and in vivo.

Cell proliferation is usually associated with the cell cycle. Current studies have shown that cell cycle can be blocked in the G0/G1 phase after treatment with morusin [24, 50]. Morusin could inhibit three RCC cell lines, 769-P, 786-O, and OSRC-2 cell, growth and migration, and disturb the cell cycle arrest in the G1 phase [24]. As we all know, the *p53* is the most common transcription factor in many human cancers. It plays an important role in the metabolism of normal cells and cancer cells, and it is often lost in cancer [51, 52]. In our study, cell cycle analysis showed that morusin inhibits the cell cycle in the G2/M phase by down-regulating CyclinB1 and CDK1, and up-regulating p53 and p21. Therefore, we guessed that morusin blocked the cell cycle in the G2/M phase through the p53/p21 axis. Previous reports have shown that morusin can induce apoptosis of a variety of cancer cells [44, 45, 53–56]. For example, morusin can induce apoptosis of pancreatic tumor cells and loss of mitochondrial membrane potential [42]. Based on these results, we performed apoptosis assay and Western blot to explore the effect of morusin on melanoma cells. The main findings in these studies are that morusin promotes apoptosis of melanoma cells by up-regulating C-PARP and C-Caspase3. Furthermore, because melanoma is prone to metastasis and has a strong ability of invasion [57–59], and studies have shown that after mulberry treatment of kidney cancer cells [24], lung cancer cells [43], cell migration ability is significantly limited, we proved that morusin could significantly inhibit the migration and invasion ability of melanoma cells. These studies suggest that morusin can not only induce cell cycle arrest and induce apoptosis, but also inhibit the migration and invasion ability of melanoma cells. Similarly, in mouse experiments, we also confirmed that morusin can inhibit tumorigenesis in mice. When selecting a mouse tumor model, we subcutaneously injected human melanoma cells into NOD/SCID immunodeficient mice, which are highly homologous, but the tumor heterogeneity may be slightly less than that in the syngeneic transplant mouse model.

Immune checkpoint inhibitor (ICI)-mediated immunotherapy can interfere with tumor and immune system functions by affecting multiple immune pathways [60]. Although immune-related adverse events may occur [61], its clinical efficacy and safety are worthy of recognition [62]. Immunotherapy mediated by it is a new direction of anti-tumor therapy for various cancers in recent years [63, 64]. As an important target for cancer therapy [65], p53 has been found to regulate IFN-γ-stimulated PD-L1 expression in melanoma [66]. In addition, clinical studies have found that the use of immunosuppressive agents can provide more durable survival benefits for patients with advanced melanoma [67, 68]. When DNA damage or stress occurs, p53 levels will increase, and it can induce cell cycle arrest and apoptosis [69–72]. In addition, no mutation of p53 gene in human melanoma A375-S2, A375-C6 cells has been reported [73, 74]. In order to further explore the role of p53 in our experiment, we successfully knocked down the p53 in A375 cells. We found that comparing with shGFP, knocking down p53 can partly rescue the cell growth inhibition, cell cycle arrest, apoptosis and the decreased ability of migration and invasion caused by morusin. Therefore, our results indicate that knocking down p53 can rescue the changes in proliferation, apoptosis and metastasis caused by morusin. Therefore, we suggest that morusin increases the expression of p53, which is a good strategy for the treatment of melanoma.

## Conclusion

Collectively, this study demonstrates that morusin can inhibit proliferation, migration, invasion, and survival of melanoma cells, through activating p53-mediated pathways, therefore providing a theoretical basis for morusin to be a potential drug for the treatment of melanoma in the future.

## Supplementary Information

**Additional file 1.**

**Additional file 2.**

**Additional file 3.**

## Data Availability

The datasets used and/or analyzed during the current study are available from the corresponding author on reasonable request.
