# Supplementary material for: Morusin shows potent antitumor activity for melanoma through apoptosis induction and proliferation inhibition
Source: BMC Cancer. 2023 Jun 29;23:602. doi: 10.1186/s12885-023-11080-1 (PMC10311746; doi:10.1186/s12885-023-11080-1)
Supplement: Supplementary file 1 — Additional file 1. [file 12885_2023_11080_MOESM1_ESM.pdf]

Supplementary Material -Original Images-WB

Figure 2

CDK1 34kDa

A375

DMSO 2μM 5μM 10μM

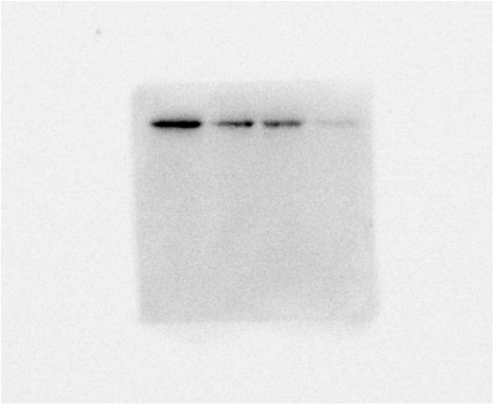

0h 12h 24h 36h

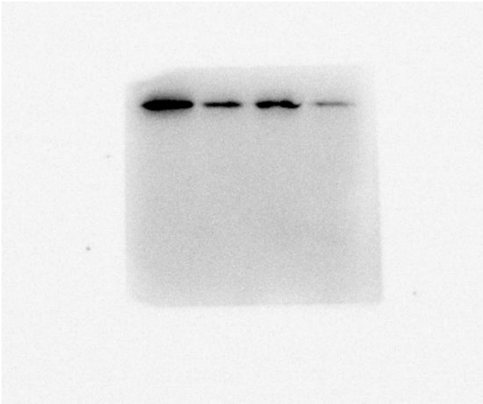

MV3

DMSO 5μM 10μM 15μM

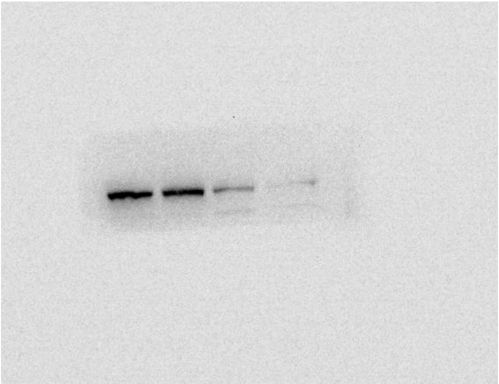

0h 12h 24h 36h

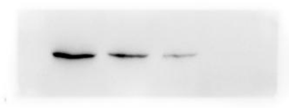

CyclinB1 55kDa

A375

DMSO 2μM 5μM 10μM

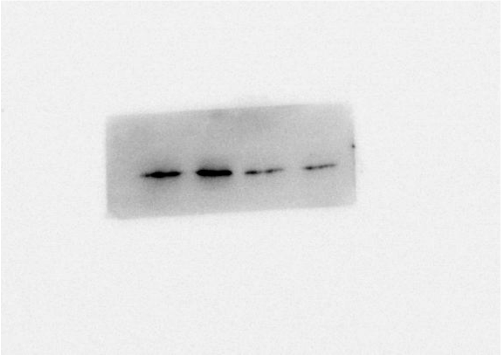

0h 12h 24h 36h

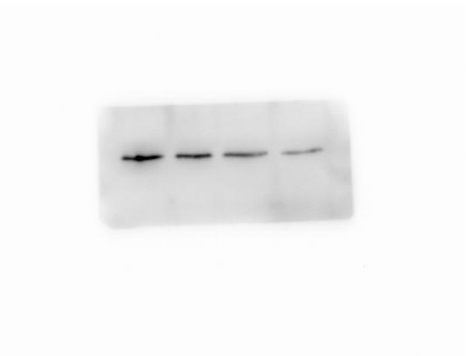

MV3

DMSO 5μM 10μM 15μM

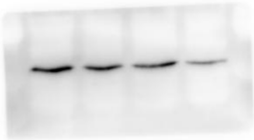

0h 12h 24h 36h

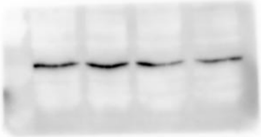

p21 21kDa

A375

DMSO 2μM 5μM 10μM DMSO 2μM 5μM 10μM(right)

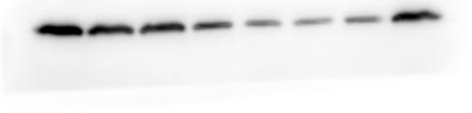

0h 12h 24h 36h 0h 12h 24h 36h(right)

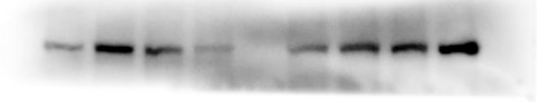

MV3

DMSO 5μM 10μM 15μM

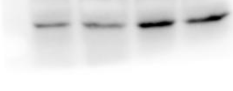

0h 12h 24h 36h

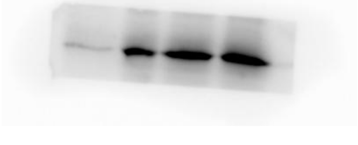

p53 53kDa

A375

DMSO 2μM 5μM 10μM

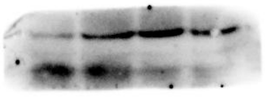

0h 12h 24h 36h

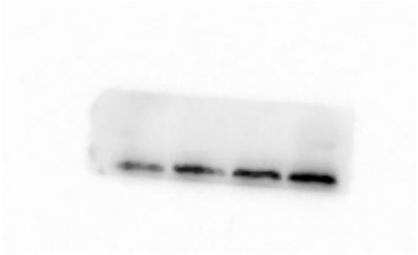

MV3

DMSO 5μM 10μM 15μM

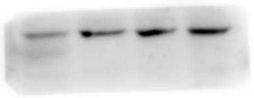

0h 12h 24h 36h

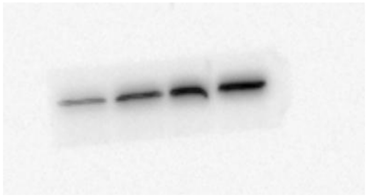

Tubulin 50kDa

A375 MV3

DMSO 2μM 5μM 10μM DMSO 5μM 10μM 15μM

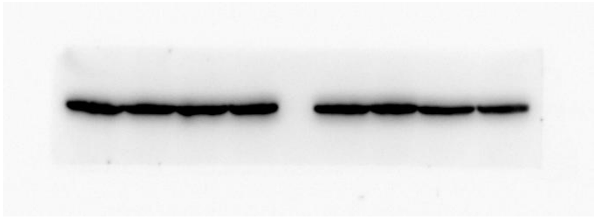

A375

DMSO 12h 24h 36h

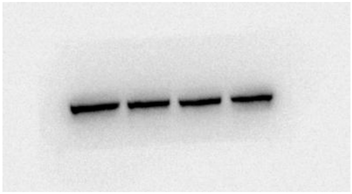

MV3

0h 12h 24h 36h

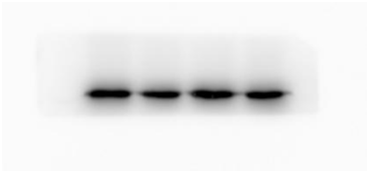

Figure 3

PARP 114-116kDa 89kDa

A375

DMSO 2μM 5μM 10μM

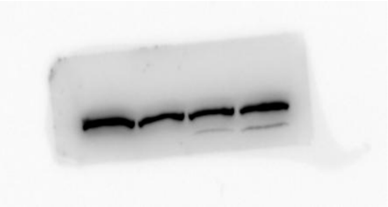

0h 12h 24h 36h

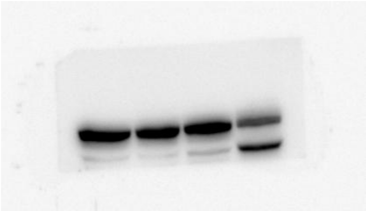

MV3

DMSO 5μM 10μM 15μM

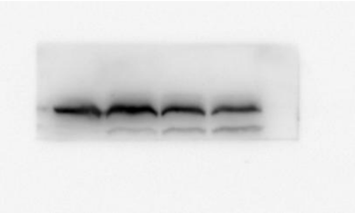

0h 12h 24h 36h

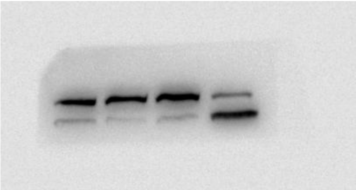

Caspase3 35kDa 17,19kDa

A375

DMSO 2μM 5μM 10μM

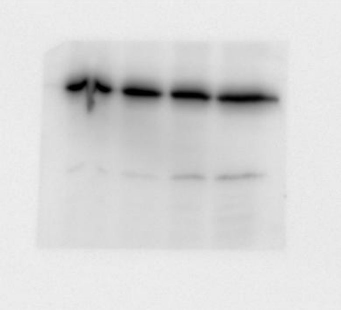

0h 12h 24h 36h

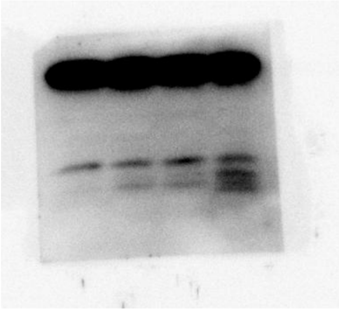

MV3

DMSO 5μM 10μM 15μM

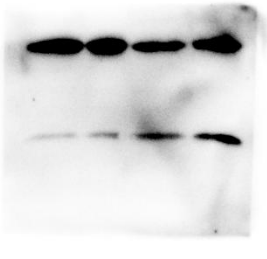

0h 12h 24h 36h

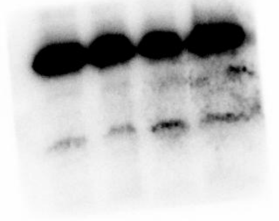

Tubulin 50kDa

A375

DMSO 2μM 5μM 10μM

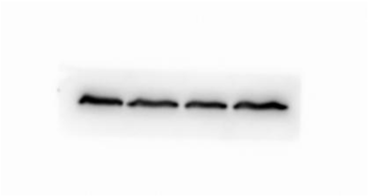

MV3

DMSO 5μM 10μM 15μM

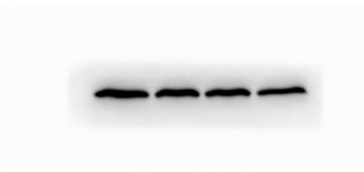

A375 MV3

0h 12h 24h 36h 0h 12h 24h 36h

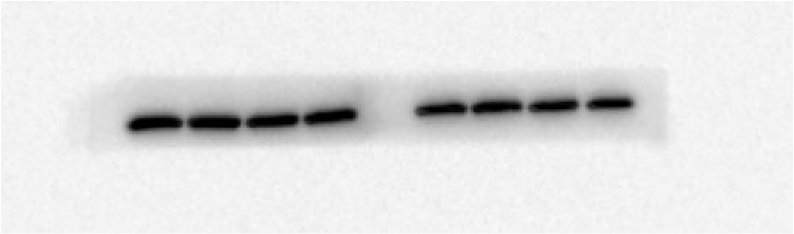

Figure 4

E-Cadherin 135kDa

A375

DMSO 2μM 5μM 10μM

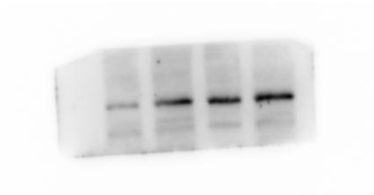

0h 12h 24h 36h

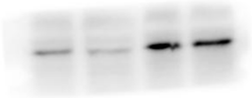

MV3

DMSO 5μM 10μM 15μM

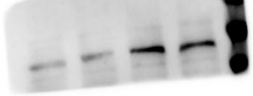

0h 12h 24h 36h(left) 0h 12h 24h 36h

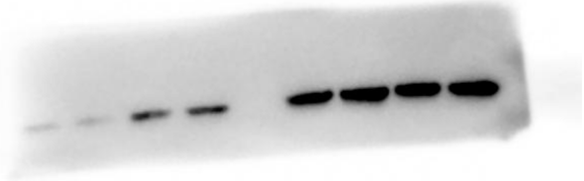

Vimentin 57kDa

A375

DMSO 2μM 5μM 10μM

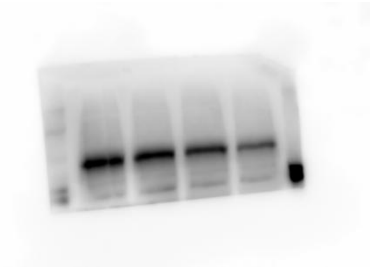

MV3

DMSO 5μM 10μM 15μM

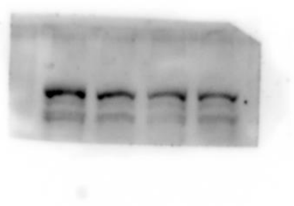

A375 MV3 MV3

0h 12h 24h 36h 0h 12h 24h 36h 0h 12h 24h 36h

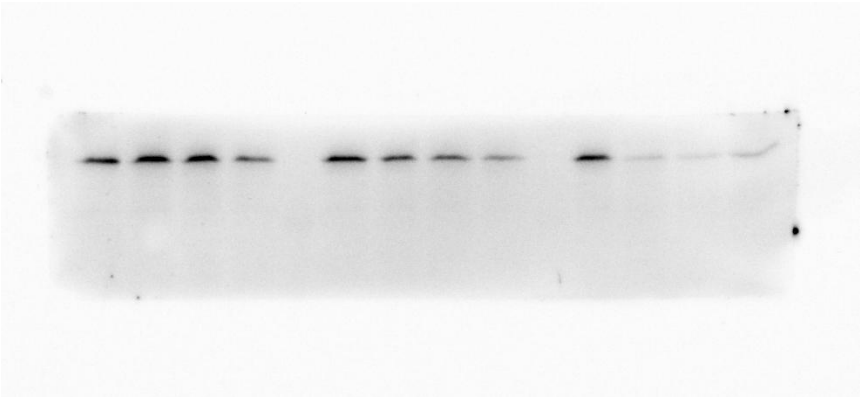

Tubulin 50kDa

A375 MV3

DMSO 2μM 5μM 10μM DMSO 5μM 10μM 15μM

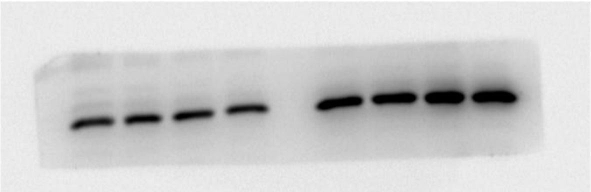

A375

0h 12h 24h 36h

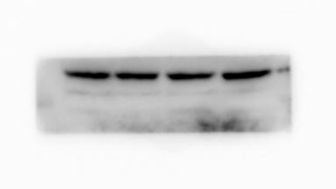

MV3

0h 12h 24h 36h

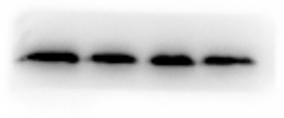

Figure 6

A p53 53kDa

MV3 A375

shGFP-shp531#-shp533# shGFP-shp531#-shp533#

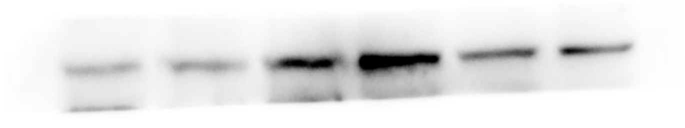

Tubulin 50kDa

MV3 A375

shGFP-shp531#-shp533# shGFP-shp531#-shp533#

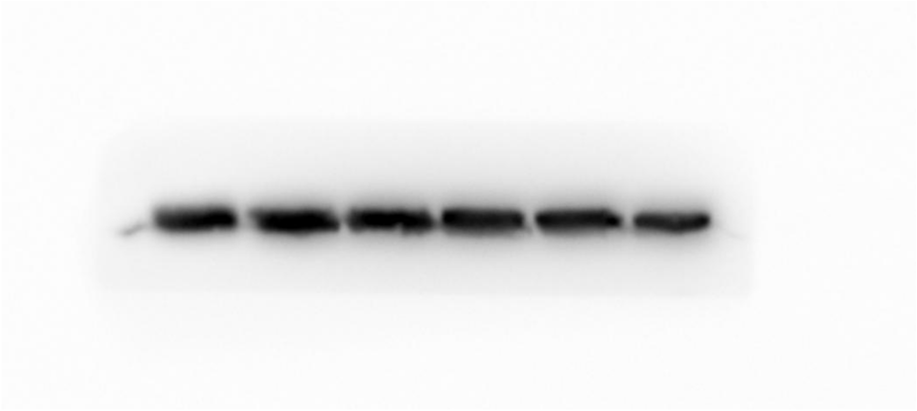

G

A375 5μM 24h

| | | | | |
|---|---|---|---|---|
| Morusin | - | + | - | + |
| Shp53 | - | - | + | + |

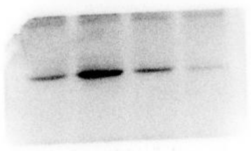

p53 53kDa

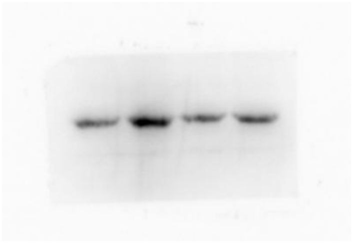

p21 21kDa

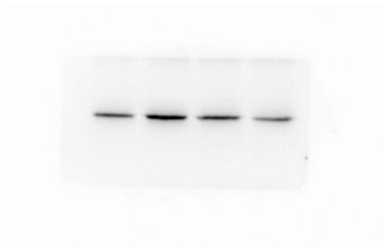

C-Caspase3 17,19kDa

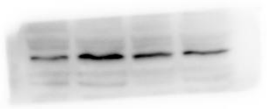

E-Cadherin 135kDa

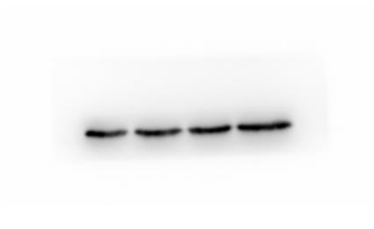

Tubulin 50kDa

S1

G

MMP2 72kDa

A375

DMSO 2μM 5μM 10μM

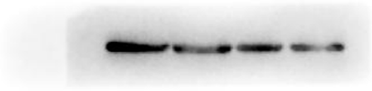

MV3

DMSO 5μM 10μM 15μM DMSO 5μM 10μM 15μM DMSO 5μM 10μM 15μM

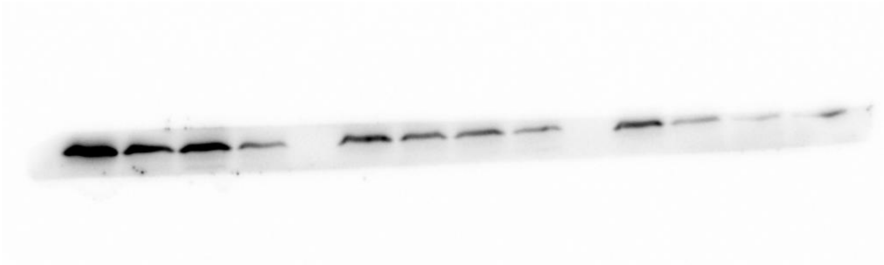

Tubulin 50kDa

A375

DMSO 2μM 5μM 10μM

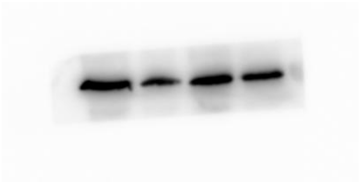

MV3

DMSO 5μM 10μM 15μM DMSO 5μM 10μM 15μM

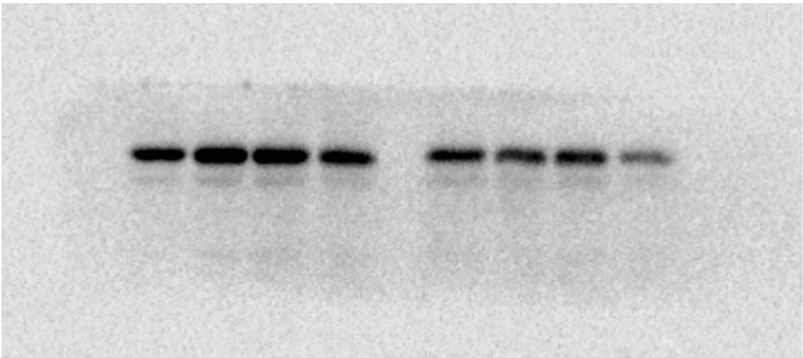

H

MMP2 72kDa

A375 MV3

0h 12h 24h 36h 0h 12h 24h 36h

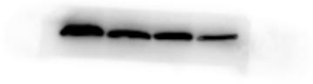

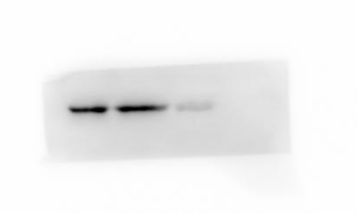

Tubulin 50kDa

A375 MV3

0h 12h 24h 36h 0h 12h 24h 36h

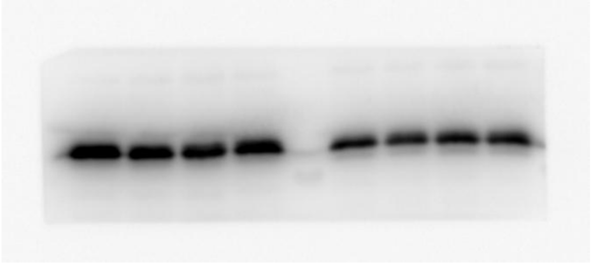

I

A375 5μM 24h

Morusin - + - +

Shp53 - - + +

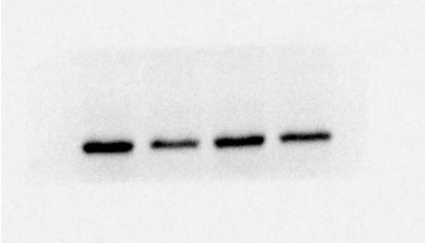

CDK1 34kDa

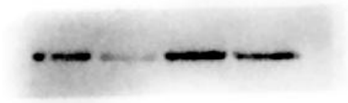

CyclinB1 55kDa

A375 5μM 24h

Morusin - + - + Morusin - + - +

Shp53 - - + + Shp53 - - + +

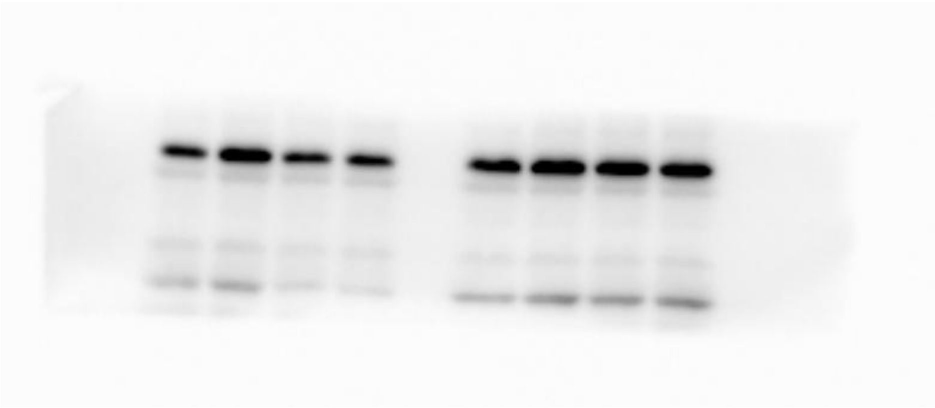

Tubulin 50kDa
