# Supplementary material for: Morusin shows potent antitumor activity for melanoma through apoptosis induction and proliferation inhibition
Source: BMC Cancer. 2023 Jun 29;23:602. doi: 10.1186/s12885-023-11080-1 (PMC10311746; doi:10.1186/s12885-023-11080-1)
Supplement: Supplementary file 2 — Additional file 2. [file 12885_2023_11080_MOESM2_ESM.docx]

**Supplementary Figures**

**Supplementary Fig. 1 A** The chemical formula structure of morusin. **B** After treating the cells with different concentrations of morusin, fluorescence quantitative PCR detects the mRNA level of p53. The effects of morusin on PIG1 and HaCaT cells. The IC50 of **C** PIG1 and **D** HaCaT cells. The viability of **E** PIG1 and **F** HaCaT after treating with DMSO or 10 μΜ morusin. **G-H** The expression levels of MMP2 in A375 and MV3 cells treated with morusin in different concentrations (2, 5, 10 μΜ for A375 and 5, 10, 15 μΜ for MV3) or DMSO for 24 h were determined by Western blot analysis. Tubulin was used as a control. **I** The expression of CyclinB1 and CDK1 were checked in knockdown p53 A375 cells treated with 5 μΜ morusin for 24 h. DMSO and shGFP were used as control. Tubulin was used as the control. **J** IC50 after DTIC treatment of A375 cells. **K** IC50 after DTIC treatment of MV3 cells. **L** The viability of A375 after treating with DMSO, morusin or DTIC. **M** The viability of MV3 after treating with DMSO, morusin or DTIC. **N** IHC staining of CDK1, CyclinB1, p53 and p21 in indicated tumors. Scale bar was 100 μm.
