# Supplementary figures and images for: Morusin shows potent antitumor activity for melanoma through apoptosis induction and proliferation inhibition
Source: BMC Cancer. 2023 Jun 29;23:602. doi: 10.1186/s12885-023-11080-1 (PMC10311746; doi:10.1186/s12885-023-11080-1)

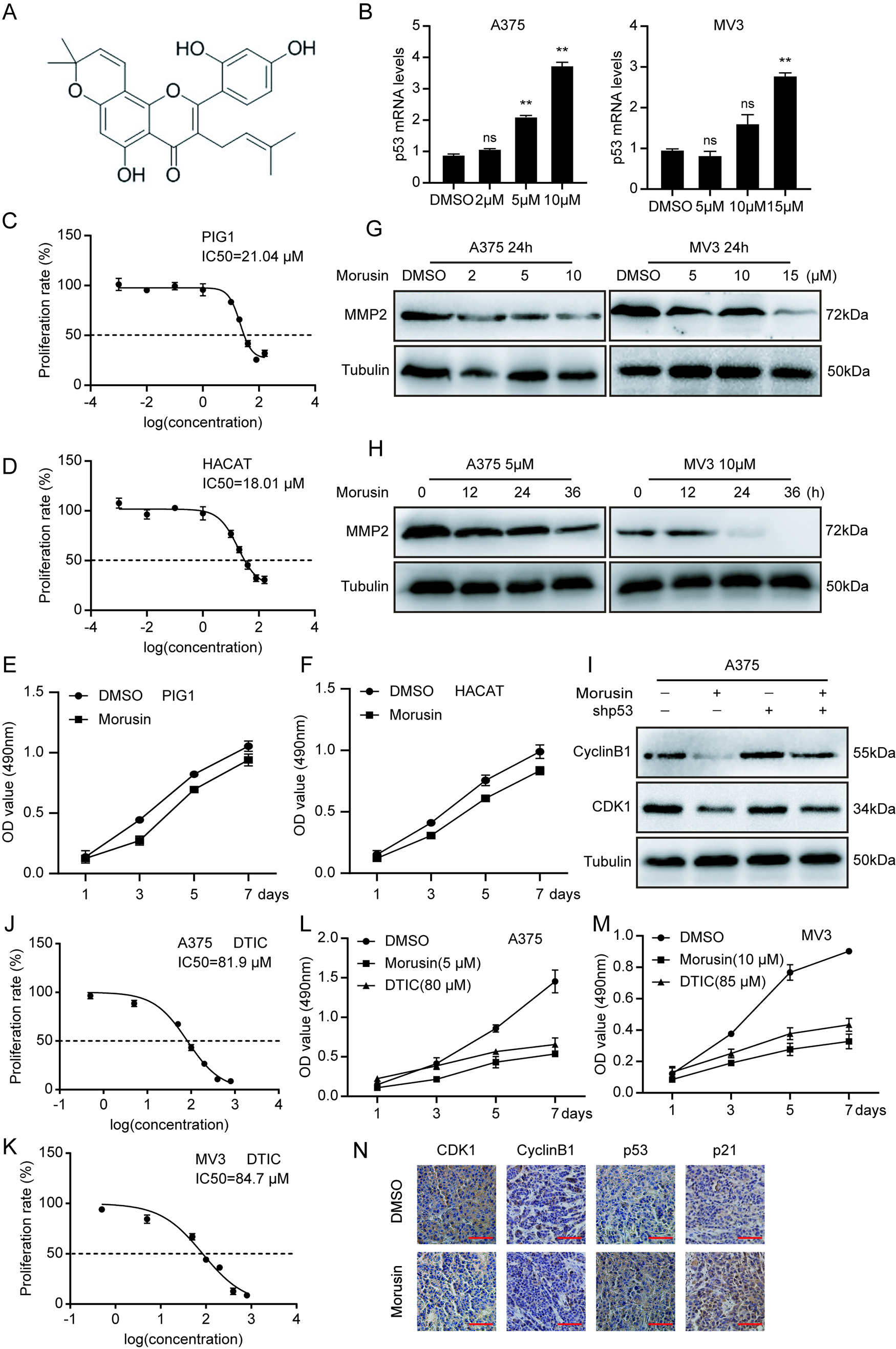

Supplement: Supplementary file 3 — Additional file 3. [file 12885_2023_11080_MOESM3_ESM.tif]
